# Risk factors of osimertinib-related cardiotoxicity in non-small cell lung cancer

**DOI:** 10.3389/fonc.2024.1431023

**Published:** 2024-07-12

**Authors:** Yunlong Wang, Xuan Deng, Qinggui Qiu, Mengchao Wan

**Affiliations:** ^1^ Department of Oncology, Jiangxi Provincial People’s Hospital, The First Affiliated Hospital of Nanchang Medical College, Nanchang, China; ^2^ Department of Outpatient, Jiangxi Provincial People’s Hospital, The First Affiliated Hospital of Nanchang Medical College, Nanchang, China

**Keywords:** osimertinib, non-small cell lung cancer, cardiotoxicity, risk factor, EGFR-TKIs

## Abstract

**Objective:**

To investigate the risk factors associated with cardiotoxicity in patients with non-small cell lung cancer (NSCLC) treated with osimertinib.

**Methods:**

A total of 268 patients with NSCLC treated with osimertinib in our hospital from June 2019 to December 2023 were selected to observe the occurrence of cardiotoxicity and were divided into cardiotoxicity group and non-cardiotoxicity group. The differences in age, gender, body mass index (BMI), smoking, alcohol consumption, tumor stage, hypertension, diabetes, hyperlipidemia, chemotherapy, radiotherapy, antiangiogenic drugs, and osimertinib treatment time were recorded and analyzed. Logistic regression was used to analyze the risk factors for cardiotoxicity in patients with non-small cell lung cancer caused by osimertinib treatment.

**Results:**

Among the 268 patients with NSCLC treated with osimertinib, 58 patients developed cardiotoxicity, and the incidence of cardiotoxicity was 21.64%. There were statistically significant differences between the cardiotoxicity group and the non-cardiotoxicity group in terms of smoking history, hyperlipidemia history, combined chemotherapy, and combined radiotherapy (P < 0.05). Further analysis showed that patients with a smoking history were at increased risk of cardiotoxicity compared with non-smoking patients (OR = 2.569, 95% CI = 1.398–6.523). Patients with hyperlipidemia were at increased risk of cardiotoxicity compared with those without hyperlipidemia (OR = 3.412, 95% CI = 2.539–7.628). Patients with chemotherapy were at increased risk of cardiotoxicity compared with those without combination chemotherapy (OR = 2.018, 95% CI = 1.426–4.517). Patients undergoing radiotherapy to the left chest were at increased risk of cardiotoxicity compared with those without combined radiotherapy (OR = 1.629, 95% CI = 1.273–4.206).

**Conclusion:**

The incidence of cardiotoxicity in patients with NSCLC is high due to osimertinib treatment. A history of smoking, hyperlipidemia, combination chemotherapy, and radiotherapy to the left chest are independent risk factors for cardiotoxicity in patients with NSCLC treated with osimertinib.

## Introduction

Lung cancer is currently one of the most common malignant tumors in the world, with 2,206,771 and 1,796,144 new cases and deaths in 2020, accounting for 11.4% (second) and 18.0% (first) of the total number of new malignant tumor cases and deaths worldwide (1). Among them, non-small cell lung cancer (NSCLC) is the most common histological type of lung cancer, accounting for approximately 85% of all cases (2). Epidemiological data from the International League of Lung Cancer showed that EGFR mutations accounted for 35.0% of 4231 NSCLC patients who underwent epidermal growth factor receptor (EGFR) gene testing (3). Osimertinib is the world’s first marketed third-generation tyrosine kinase inhibitor (TKI). The FLAURA study suggests that osimertinib can prolong the survival of NSCLC patients, and its common adverse effects include diarrhea, rash, paronychia, dry skin, and oral mucositis (4). Osimertinib-related cardiotoxicity is continuously rising cardiac abnormalities when patients are treated with osimertinib (5). The overall risk of osimertinib inducing serious (grade >3) adverse effects was lower than that of gefitinib or erlotinib (42% vs. 47%), but the incidence of cardiotoxicity was relatively higher (reduced left ventricular ejection fraction: 5% vs.2%; QT interval prolongation by 10% vs.4%) (6, 7).

Osimertinib has excellent antitumor performance, but its potential cardiotoxicity after treatment limits its wider clinical application (8). In addition, the toxic effects of osimertinib on the heart are diverse and the onset is hidden, difficult to detect, and irreversible, which seriously affects the quality of life of cancer patients and even endangers the lives of patients (9). Clinically, a significant proportion of cancer patients die not from tumors but from subsequent cardiac complications. Therefore, early diagnosis and early prevention are extremely important for the prevention and treatment of cardiotoxicity (10). Therefore, early detection of high-risk factors for osimertinib-related cardiotoxicity is a key link in the prevention and treatment of osimertinib-related cardiotoxicity. In this manuscript, we describe risk factors of osimertinib-related cardiotoxicity in NSCLC.

## Materials and methods

### Subject

This study is a single-center, retrospective, observational study conducted in Jiangxi Provincial People’s Hospital. The records of NSCLC patients with EGFR mutations received osimertinib targeted anticancer therapy were retrieved through our medical record system. Eventually, a total of 268 patients with advanced NSCLC treated with osimertinib were enrolled in the study. All patients have signed the informed consent form, and this study has been reviewed by the medical ethics committee of our hospital.

### Inclusion criteria

The inclusion criteria are as follows: (1) patients with histologically and cytologically confirmed advanced NSCLC; (2) be over 18 years old and under 90 years old; (3) the treatment regimen is osimertinib monotherapy or osimertinib in combination with other chemotherapy drugs (platinum-containing dual-drug regimen chemotherapy) (excluding anthracyclines); (4) electrocardiogram (ECG, including QT interval) was normal before targeted drug therapy; (5) BNP, myocardial injury markers, and left ventricular ejection fraction within normal limits before starting treatment.

### Exclusion criteria

The exclusion criteria are as follows: (1) lack of ECG data at least once before and after medication; (2) duration of osimertinib for <2 weeks; (3) combined with coronary heart disease, including after cardiac vascular stent implantation and cardiac coronary artery bypass grafting; (4) patients whose ECG is not suitable for QT interval measurement, including atrial fibrillation, atrioventricular block of grade II and above, pacemaker or implantable cardioverter-defibrillator implantation, multiple premature ventricular contractions, multiple premature atrial contractions, preexcitation syndrome, and sick sinus syndrome; (5) are taking medications known to prolong the OT interval (e.g., anthracyclines); (6) patients with severe infection, renal failure on dialysis treatment, and immune diseases.

### Methodology

Osimertinib was taken according to the instructions, the chemotherapy regimen was in line with the CSCO guidelines, and the specific dosage of each drug was calculated according to the body surface area. Radiotherapy depends on the condition, and the dose and fraction of radiotherapy are based on the actual situation.

Diagnostic criteria for cardiotoxicity: (1) cardiomyopathy with reduced left ventricular ejection fraction (LVEF), manifested by decreased overall function or significantly reduced ventricular septal motion; (2) symptoms associated with congestive heart failure (CHF); (3) CHF-related signs, such as gallop rhythm of the third heart sound, tachycardia, or both; (4) LVEF is reduced by at least 5% to <55% absolute from baseline, with symptoms of CHF, or LVEF is reduced by at least 10% to <55% absolute, with no symptoms or signs of CHF [8]. The enrolled patients were divided into groups according to the presence or absence of cardiotoxicity, which were cardiotoxicity group (n = 58) and no cardiotoxicity group (n = 210). The age, gender, body mass index (BMI), smoking, alcohol consumption, tumor stage, hypertension, diabetes, hyperlipidemia, chemotherapy, radiotherapy, antiangiogenic drugs, osimertinib treatment time, and other clinical data were recorded.

### Statistical methods

With SPSS 20. 0. Software statistical analysis data. Count data are expressed in cases or percent. Univariate analysis was used to perform chi-square test or Fisher test, and then according to the results, the statistically significant indicators were used as the independent variable, the occurrence of cardiotoxicity was used as the dependent variable, and the logistic regression analysis was performed. P < 0. 05 statistically significant for the difference.

## Results

### Incidence of cardiotoxicity

A total of 268 patients with non-small cell lung cancer were treated with osimertinib, of which 58 developed cardiotoxicities, with a cardiotoxicity rate of 21.64%. The incidence of QT interval prolongation, LVEF decrease, heart failure, cardiac tamponade, myocardiopathy, supraventricular tachycardia, myocardial infarction, and cardiac arrest is 39 (10.6%), 20 (5.4%), 4 (1.1%), 6 (1.6%), 7 (1.9%), 6 (1.6%), 5 (1.4%), and 4 (1.1%), respectively.

### Analysis of influencing factors of cardiotoxicity

There were significant differences between the cardiotoxic group and the non-cardiotoxic group in terms of smoking history, hyperlipidemia history, combined chemotherapy, and left thoracic radiotherapy history (P < 0.05). There were no significant differences in age, gender, body mass index (BMI), alcohol consumption, tumor stage, history of hypertension, history of diabetes mellitus, combination of anti-angiogenic drugs, and duration of osimertinib treatment between the two groups (P > 0.05), as shown in **Table 1**.

**Table 1:** Characteristics of the patients with and without cardiotoxicity (n = 268).

Variable		Cardiotoxicity group (N=58) n (%)	Non-Cardiotoxicity group (N=210) n (%)	x^2^	*p*
Age				0.095	0.446
	< 60 years	17 (20.5)	66 (79.5)		
	≥ 60 years	41 (22.2)	144 (77.8)		
Gender				0.324	0.337
	Male	26 (20.2)	103 (79.8)		
	Female	32 (23.0)	107 (77.0)		
**BMI (kg/m2)**				0.102	0.992
	< 18.5	2 (18.2)	9 (81.8)		
	18.5~24	23 (21.5)	84 (78.5)		
	24~28	29 (22.1)	102 (77.9)		
	≥28	4 (21.1)	15 (78.9)		
Smoking				14.226	0.000^*^
	current	41 (30.8)	92 (69.2)		
	former	13 (15.5)	71 (84.5)		
	No	4 (7.8)	47 (92.2)		
Excessive alcohol consumers				1.510	0.140
	Yes	37 (24.3)	115 (75.7)		
	No	21 (18.1)	95 (81.9)		
Clinical stage				3.373	0.338
	I	2 (16.7)	10 (83.3)		
	II	15 (21.1)	56 (78.9)		
	III	26 (19.0)	111 (81.0)		
	IV	15 (31.3)	33 (68.7)		
Hypertension				0.804	0.227
	Yes	31 (19.7)	126 (80.3)		
	No	27 (24.3)	84 (75.7)		
Diabetes				1.256	0.166
	Yes	36 (24.2)	113 (75.8)		
	No	22 (18.5)	97 (81.5)		
Hyperlipidemia				5.231	0.016^*^
	Yes	38 (27.1)	102 (72.9)		
	No	20 (15.6)	108 (84.4)		
Chemotherapy				3.680	0.037^*^
	Yes	42 (25.5)	123 (74.5)		
	No	16 (15.5)	87 (84.5)		
Thoracic radiotherapy				6.247	0.045^*^
	Left	32 (29.1)	78 (70.9)		
	right	21 (15.9)	111 (84.1)		
	No	5 (19.2)	21 (80.8)		
Antiangiogenic drugs				0.111	0.427
	Yes	39 (21.1)	146 (78.9)		
	No	19 (22.9)	64 (77.1)		
Treatment time of Osimertinib				0.161	0.984
	< 1 year	4 (19.0)	17 (81.0)		
	2~3 years	13 (21.0)	49 (79.0)		
	3~4 years	21 (21.6)	76 (78.4)		
	≥4 years	20 (22.7)	68 (77.3)		

BMI, body mass index, *p<0.05. The bold values means the P value is less than 0.05.

### Multivariate logistic regression analysis

The factors of P < 0.05 screened in the univariate analysis were used as independent variables, including smoking history (no smoking = 0, quit smoking = 1, smoking = 2; the dumb variable was set, taking no smoking as the reference), history of lipidemia (no history of hyperlipidemia = 0, history of hyperlipidemia = 1), combination chemotherapy (no chemotherapy = 0, chemotherapy = 1, dumb variables, with no chemotherapy as a reference), and combination with radiotherapy (no radiotherapy = 0, right chest radiotherapy = 1, left thoracic radiotherapy = 2; set the dummy variable, with no radiotherapy as the reference); dichotomous logistic regression analysis was performed with the occurrence of cardiotoxicity as the dependent variable (no cardiotoxicity = 0, occurrence of cardiotoxicity = 1). The results showed an increased risk of cardiotoxicity in patients with a history of smoking (OR = 2.569, 95% CI = 1.398–6.523). Patients with hyperlipidemia were at increased risk of cardiotoxicity compared with those without hyperlipidemia (OR = 3.412, 95% CI = 2.539–7.628). Patients with chemotherapy were at increased risk of cardiotoxicity compared with those without combination chemotherapy (OR = 2.018, 95% CI = 1.426–4.517). Patients with radiotherapy to the left chest were at increased risk of cardiotoxicity compared with patients without combined radiotherapy (OR = 1.629, 95% CI = 1.273–4.206), as shown in **Table 2**.

**Table 2:** Multiple logistic regression analysis of influencing factors of osimertinib - related cardiotoxicity.

Factors	*β*	S.E.	Wald	df	Exp(B)	95% C.I.for Exp(B)	Sig.
Univariate							
Age	0.339	0.867	1.794	1	0.823	0.257–7.673	0.696
Gender	1.243	1.642	3.458	1	0.914	0.926–6.513	0.457
BMI	0.466	1.863	1.492	1	0.627	0.348–4.271	0.589
Hypertension	1.323	1.029	1.537	1	0.725	0.623–3.819	0.268
Diabetes	1.465	1.712	2.537	1	0.993	0.517–5.432	0.721
Smoking	3.526	0.852	4.618	1	1.457	1.347–2.963	0.029*
Hyperlipidemia	2.242	0.768	3.397	1	2.713	2.198–4.629	0.001*
Chemotherapy	1.516	0.628	2.849	1	2.365	1.426–5.193	0.003*
Thoracic radiotherapy	1.626	0.724	2.658	1	1.813	1.037–3.263	0.048*
Multivariate							
Smoking	1.152	0.437	6.729	1	2.569	1.398–6.523	0.008
Hyperlipidemia	1.580	0.693	3.527	1	3.412	2.539–7.628	0.000*
Chemotherapy	0.723	0.268	5.146	1	2.018	1.426–4.517	0.016*
Thoracic radiotherapy	0.916	0.417	2.238	1	1.629	1.273–4.206	0.036*

*p<0.05.

## Discussion

As far as we know, our study is the first to investigate the risk factors of osimertinib-induced cardiotoxicity in patients with NSCLC. Our study shows that the osimertinib-related cardiotoxicity rate is 21.64%. Moreover, the smoking history, hyperlipidemia history, combination chemotherapy, and combination radiotherapy to the left chest are independent risk factors for osimertinib-related cardiotoxicity. Osimertinib-related cardiotoxicity is a type II cancer treatment-related cardiac dysfunction (CTRCD), which may have serious consequences, but myocardial damage is generally reversible, so early diagnosis and timely intervention are particularly important (11, 12). It is recommended that clinicians should conduct a baseline risk assessment of patients before starting osimertinib, including previous history (e.g., hypothyroidism, interstitial lung disease, or heart disease), past history (e.g., history of chest radiation therapy), and family history (e.g., long QT syndrome).

Smoking increases the hazard of the cardiovascular system, even sudden death (13, 14). Smoking also effects the nitric oxide (NO) reduction and leads to vasomotor dysfunction, pro-thrombogenic effects, and alteration of lipid metabolism (increase in oxidative LDL) and induces inflammation and oxidative stress (14). Smoking significantly increases the risk of hypertension and insulin resistance, which gradually facilitate the development of cardiovascular diseases (15). Smoking mainly damages endothelial cells and leads to side effects (16). Therefore, smoking may act as a synergy and as a risk factor osimertinib-related cardiotoxicity.

During osimertinib therapy, attention should be paid to the patient’s combination of medications (e.g., moxifloxacin, bevacizumab, or granisetron) and to the possible harm caused by drug interactions. Healthcare providers should focus on patients with these risk factors and promptly monitor biochemical markers (e.g., B-type brain natriuretic peptide, troponin, myoglobin, and electrolytes) and imaging markers [e.g., ECG, echocardiography, or magnetic resonance imaging (MRI)] that suggest cardiac dysfunction (17, 18). Similarly, radiotherapy, as an effective antitumor treatment for lung cancer patients, may have a synergistic effect with the combination of osimertinib, leading to an increased cardiotoxicity. The results of our study suggest that radiotherapy may increase the risk of cardiotoxicity by 1.629 times. Risk of developing cardiovascular toxicity after RT is closely linked to the mean heart dose (MHD), a reflection of cardiac radiation exposure, and also depends on dose distribution and exposure of specific cardiac substructures. Generally, >15 Gy to 25 Gy MHD is considered high risk, and >25 Gy MHD confers very high risk (19).

In our study, we found that hyperlipidemia is one of the risk factors of osimertinib-induced cardiotoxicity in patients with NSCLC. Previous studies have shown that hyperlipidemia was one of the highly prevalent cardiovascular risk factors among lung cancer patients (20). Furthermore, statin initiation is currently recommended in primary prevention for patients with atherosclerotic cardiovascular disease (21). Therefore, hyperlipidemia is one of the main factors to avoid osimertinib-induced cardiotoxicity in lung cancer patients.

Our study is a monocentric study, so there are many limitations. The small number of patients, nationality, and diagnostic criteria of cardiotoxicity may affect the incidence of osimertinib-induced cardiotoxicity in NSCLC.

Osimertinib plays an important role in the treatment of NSCLC as a representative drug of the third-generation EGFR-TKIs (22). Due to the large population base of lung cancer and the wide clinical application of osimertinib, the reports of cardiotoxicity have been increasing in recent years and may have serious consequences, and medical personnel should pay full attention to it, especially when patients have risk factors such as heart disease, electrolyte imbalance, hypothyroidism, or inappropriate drugs. In addition, as a targeted preparation for oral administration, osimertinib is self-administered outside the hospital to increase the risk of medication for patients, and clinicians and pharmacists should inform patients of the possibility of osimertinib inducing cardiac injury, strengthen drug education, and advise them to undergo regular cardiac function tests to ensure drug safety.

## Data availability statement

The original contributions presented in the study are included in the article/supplementary material. Further inquiries can be directed to the corresponding author.

## Ethics statement

The studies involving humans were approved by the medical ethics committee of Jiangxi Provincial People’s Hospital. The studies were conducted in accordance with the local legislation and institutional requirements. Written informed consent for participation in this study was provided by the participants’ legal guardians/next of kin.

## Author contributions

YW: Conceptualization, Investigation, Methodology, Project administration, Writing – review & editing. XD: Data curation, Formal analysis, Software, Writing – review & editing. QQ: Data curation, Formal analysis, Investigation, Writing – review & editing. MW: Conceptualization, Funding acquisition, Methodology, Project administration, Resources, Supervision, Writing – original draft, Writing – review & editing.
